# AI-Based Detection of Osteoporosis on Dental Radiographs: Influence of Region-of-Interest Selection on Classification Performance

**DOI:** 10.3390/jimaging12070285

**Published:** 2026-06-27

**Authors:** Michael Moncher, Vincent Traboulsi, Florian Kofler, Sarah Müller, Felix Steinbauer, Constantin von See

**Affiliations:** 1 Research Center for Digital Technologies in Dentistry and Computer Aided Design/Computer-Aided Manufacturing, Department of Dentistry, Faculty of Medicine and Dentistry, Danube Private University, Steiner Landstraße 124, 3500 Krems an der Donau, Austria; traboulsi.vincent@dp-uni.eu (V.T.); constantin.vonsee@dp-uni.ac.at (C.v.S.); 2 Department of Quantitative Biomedicine, University of Zurich, Winterthurerstrasse 190, 8057 Zurich, Switzerland; florian.kofler@uni-tuebingen.de; 3 Helmholtz AI, Helmholtz Munich (German Research Center for Environmental Health), Ingolstädter Landstrasse 1, 85764 Neuherberg, Germany; 4 AI for Image-Guided Diagnosis and Therapy, TUM School of Medicine and Health, Technical University of Munich, Ismaninger Strasse 22, 81675 Munich, Germany; 5 Hertie Institute for AI in Brain Health, University of Tübingen, Otfried-Müller-Strasse 27, 72076 Tübingen, Germany; sar.mueller@uni-tuebingen.de; 6 Tübingen AI Center, University of Tübingen, Otfried-Müller-Strasse 27, 72076 Tübingen, Germany; 7 School of Computation, Information and Technology, Technical University of Munich, Boltzmannstrasse 3, 85748 Garching b. München, Germany; felix.steinbauer@tum.de; 8 Munich Center for Machine Learning (MCML), Technical University of Munich, Boltzmannstrasse 3, 85748 Garching b. München, Germany

**Keywords:** osteoporosis, dental radiography, artificial intelligence, deep learning, peri-implant bone, mental foramen, ResNet, medical imaging, radiographic classification

## Abstract

Osteoporosis may alter mandibular bone structure and peri-implant remodeling, but it remains unclear whether such changes are detectable on dental radiographs using deep learning. This retrospective study evaluated whether osteoporosis can be discriminated in two mandibular regions of interest: peri-implant bone and the mental foramen region. Digital periapical radiographs acquired between November 2012 and October 2024 were analyzed in 51 women, including 25 patients with osteoporosis and 26 non-osteoporotic controls without a documented history or diagnosis of osteoporosis; the osteoporosis group was significantly older than the control group. Two binary classification experiments were performed using patient-level fivefold grouped cross-validation. The peri-implant experiment included 1682 cropped images and used an image-plus-metadata ResNet-18 model incorporating the time interval between implant placement and radiograph acquisition. The mental foramen experiment included 102 cropped images and used an image-only ResNet-18 model. Mean accuracy, F1 score, and area under the receiver operating characteristic curve were 0.613, 0.628, and 0.713 for the peri-implant region of interest (ROI) and 0.701, 0.713, and 0.744 for the mental foramen ROI, respectively. Both experiments showed substantial fold-to-fold variability. These findings suggest that ROI selection influences model behavior, but neither approach yielded sufficiently stable ROI-level classification performance under patient-level grouped validation to support individual patient-level screening claims. Nondiscriminatory AI results should therefore be interpreted as limited evidence under the present experimental conditions rather than as proof of radiographic equivalence.

## 1. Introduction

Artificial intelligence (AI) has become an important component of medical imaging research because it can identify complex image patterns that may be difficult to detect by visual inspection alone. In dental radiology, AI has already been applied to the detection of caries, periapical lesions, periodontal bone loss, and anatomic structures, with promising but heterogeneous results across tasks, datasets, and imaging modalities [1,2,3,4,5,6]. More recently, interest has expanded toward the possibility that dental radiographs may also contain latent signatures of systemic disease, including osteoporosis [7,8].

This question is biologically plausible. Osteoporosis is characterized not only by reduced bone mineral density but also by impaired bone quality, altered trabecular organization, and disturbed remodeling dynamics [9]. Experimental evidence further suggests compromised regenerative capacity in osteoporotic bone [10]. In the context of dental implants, this may be particularly relevant because implant placement creates a controlled bone injury followed by inflammation, resorption, woven bone formation, and maturation. If systemic osteoporosis affects this cascade, peri-implant bone healing could exhibit subtle radiographic differences. At the same time, osteoporosis-related alterations may also be present in more stable mandibular regions that are not influenced by surgery, such as the mental foramen region. Previous osteoporosis-screening studies using panoramic radiographs have shown that mandibular cortical and trabecular features can carry diagnostically relevant information, and computer-aided approaches have already been explored in this setting [11,12,13,14].

However, the central challenge is not only biological, but methodological. In AI studies, failure to discriminate between clinical groups does not demonstrate that no radiographic difference exists. As emphasized in classical statistics, absence of evidence is not evidence of absence [15]. In medical imaging AI, weak or unstable performance may instead arise from limited sample size, fold instability, hidden confounding, shortcut learning, poor generalizability, or uncertainty related to model and data limitations [16,17,18,19,20,21]. This problem is especially relevant in small radiologic datasets, where internal performance may be misleading and negative findings are often difficult to interpret rigorously [22,23,24,25]. Formal equivalence concepts may therefore be more appropriate than conventional significance logic when the scientific question concerns indistinguishability rather than superiority [26,27,28].

The hypothesis of this study was that osteoporosis-related radiographic signatures, if present, would differ between a peri-implant region of interest (ROI), representing bone undergoing healing and re-ossification, and a mental foramen ROI, representing more stable mandibular bone architecture. Accordingly, the purpose of this study was twofold: first, to evaluate whether osteoporosis can be discriminated on dental radiographs in these two biologically distinct ROIs, and second, to examine how nondiscriminatory AI results in dental imaging should be interpreted methodologically [8,29,30].

## 2. Materials and Methods

### 2.1. Study Design and Study Population

This retrospective comparative study used medical imaging data for binary image classification. The dataset comprised digital periapical radiographs retrieved from an institutional archive. The study population consisted of 51 female patients and was divided into two groups: an osteoporosis group (*n* = 25) and a control group (*n* = 26). All patients in the osteoporosis group had a confirmed diagnosis of osteoporosis based on dual-energy X-ray absorptiometry (DXA), using a T-score of −2.5 or lower as the diagnostic threshold. The same diagnostic threshold was applied consistently to all patients in the osteoporosis group. Patients in the control group had no documented history or diagnosis of osteoporosis. Information on osteoporosis-related medication, including antiresorptive or anabolic therapy, was reviewed when available. Disease severity and disease duration were also considered in the clinical data review; however, because these variables were not uniformly documented across all patients, they were not included as covariates in the model. This limitation was considered when interpreting the classification results. The radiographs included in this study were acquired between November 2012 and October 2024. The study protocol was approved by the Lower Austria Ethics Review Committee (GS4-EK-4/379-2016). Owing to the retrospective study design and the use of anonymized archived radiographic data, the requirement for informed consent was waived by the ethics committee.

Two separate image-classification experiments were performed to evaluate whether osteoporosis-related radiographic differences could be detected in two mandibular regions of interest (ROIs): peri-implant bone and the mental foramen region. Both experiments used the same patient groups, class labels, and patient-level cross-validation strategy, while differing in anatomical focus and model input structure. Images were assigned to two target classes, osteoporotic (OP) and non-osteoporotic (NonOP). No additional patient-level information was used except for the time interval between implant placement and radiograph acquisition (delta-days), which was incorporated only in the peri-implant experiment.

The study focused exclusively on patients restored with implant systems from BEGO, Straumann, Biomet 3i, or Thommen. These different implant systems may introduce heterogeneity in peri-implant radiographic appearance due to differences in implant geometry, surface characteristics, prosthetic rehabilitation, and follow-up timing. This heterogeneity was considered a potential source of noise and possible shortcut learning in the peri-implant ROI experiment. To reduce the risk of patient-level information leakage and shortcut learning, all analyses were performed with patient-level grouped validation rather than image-level random splitting. This design was chosen to support a more generalizable evaluation of model performance and to avoid overinterpretation of weak or unstable discriminatory performance.

### 2.2. Dataset Construction

Radiographic samples were organized into two directories corresponding to the two class labels, OP and NonOP. These labels were used directly as the target classes for model training and validation. Each image filename contained a patient-specific identifier linking multiple radiographs to the same individual. This identifier was used to preserve patient-level grouping during cross-validation. For traceability and reproducibility, filenames also encoded additional metadata, including patient ID, jaw region (maxilla or mandible), acquisition date, and implant manufacturer.

### 2.3. Regions of Interest

The peri-implant dataset consisted of cropped image regions surrounding the implant body and adjacent alveolar bone. These crops included the peri-implant trabecular pattern and visible cortical outlines. Each ROI was resized to a standardized input dimension of 224 × 224 pixels. For this experiment, a numerical metadata variable representing the number of days between implant placement and radiograph acquisition (delta-days) was available and incorporated into the model. Because peri-implant bone is influenced by healing and remodeling, this ROI was expected to show greater biologic and radiographic variability. Accordingly, weak discriminatory performance in this region was interpreted cautiously and not assumed to indicate true equivalence between osteoporotic and non-osteoporotic bone.

**Figure 1:**
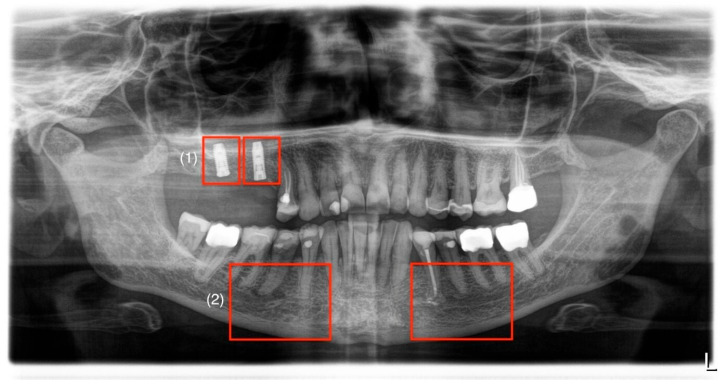
Panoramic radiograph (OPTG) illustrating the standardized regions of interest (ROIs) for systematic image extraction. Two anatomical ROIs were defined: (1) peri-implant regions around dental implants and (2) bilateral mental foramina. Red rectangles delineate these ROIs, which were digitally cropped and resized for quantitative morphological assessment.

For the second experiment, cropped ROIs including the mental foramen and the surrounding trabecular bone were used. These ROIs were selected manually to represent a comparatively stable anatomical region not directly affected by implant placement or peri-implant healing. All foramen ROIs were resized to the same standardized image dimensions used in the peri-implant experiment. Compared with peri-implant bone, the foramen region was considered anatomically more stable and therefore potentially more suitable for assessing systemic osteoporosis-related bone characteristics. The anatomical ROI definition and standardized extraction strategy are illustrated in Figure 1.

### 2.4. Image Preprocessing

All images underwent a uniform preprocessing pipeline. When required, images were converted to grayscale. Contrast-limited adaptive histogram equalization was applied to standardize local contrast [31]. Images were then resized to a fixed spatial resolution and normalized using standard intensity scaling. During training, data augmentation was applied to improve robustness to imaging variability and included random rotations, flips, and brightness or contrast adjustments. Augmentations were applied only to training images and not to validation images.

### 2.5. Model Architectures

The peri-implant experiment used a convolutional neural network based on ResNet-18 [32]. The architecture processed the image ROI through a convolutional backbone and, in parallel, processed the normalized delta-days variable through a metadata pathway. The image-derived feature representation and metadata feature were fused before the final classification layer. The model output consisted of two logits corresponding to the OP and NonOP classes.

The foramen experiment used an image-only ResNet-18-based classifier [32]. This model consisted of the convolutional backbone, a global feature aggregation stage, and fully connected classification layers producing two output logits for the OP and NonOP classes.

The use of two related but distinct model configurations was intended to assess whether nondiscriminatory results might be attributable to model-input design rather than to complete absence of radiographic signal.

### 2.6. Training Procedure

**Figure 2:**
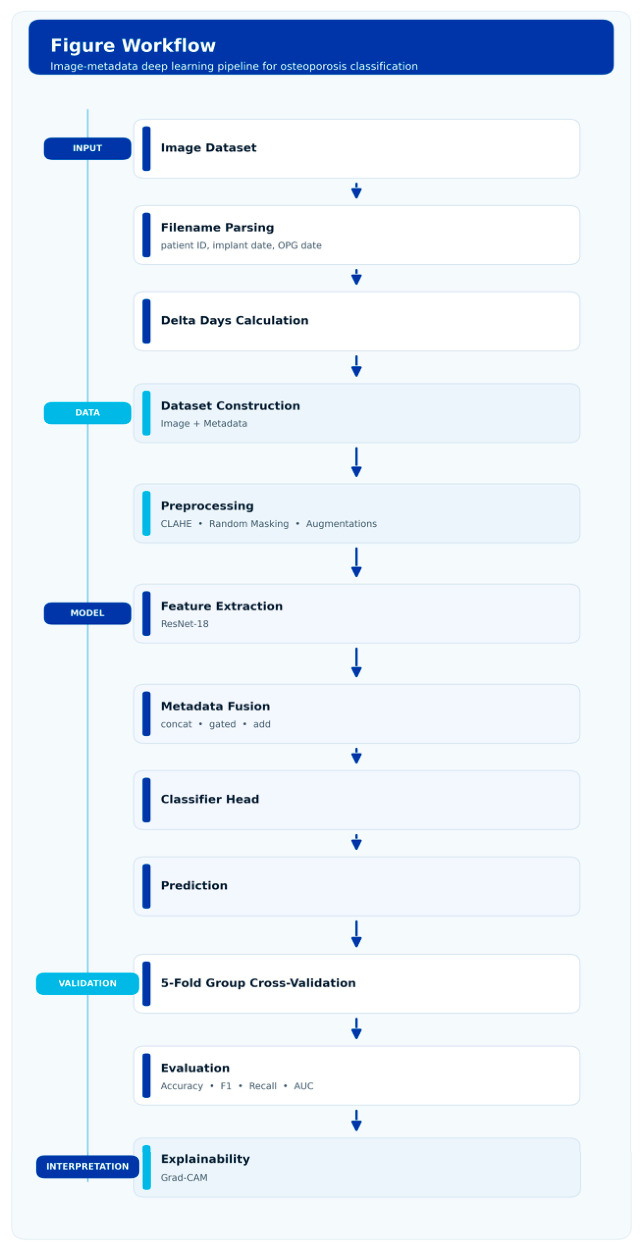
Workflow of the image-metadata deep learning pipeline. The workflow illustrates the sequential processing steps from image dataset input and filename-based metadata extraction to preprocessing, feature extraction, metadata fusion, classification, fivefold group cross-validation, performance evaluation, and Grad-CAM-based explainability analysis.

Both experiments used the same training settings. The loss function was categorical cross-entropy, the optimizer was Adam, and the learning rate was set to 0.001. Each model was trained for 50 epochs using a batch size of 16. Model weights were reinitialized at the beginning of each fold to ensure independence between cross-validation runs. During training and validation, performance metrics including accuracy, precision, recall, F1 score, and area under the receiver operating characteristic curve (AUC) were recorded. No early stopping criterion was applied. The complete image-processing, metadata-extraction, model-training, validation, and explainability workflow is summarized in Figure 2.

### 2.7. Cross-Validation

Fivefold cross-validation was performed using patient-level grouped splitting. All images from a given patient were assigned to the same fold, thereby preventing leakage of patient-specific information between training and validation sets. Each fold therefore represented a patient-separated training and validation split. In each fold, approximately 80% of the patients were used for training and 20% for validation. Thus, every patient contributed to validation exactly once across the five folds, and no separate independent test set was defined. Final performance measures were summarized across folds as mean values with corresponding standard deviations.

Because multiple image crops could originate from the same patient, the number of ROI samples was considerably larger than the number of statistically independent patients. Therefore, patient-level grouping was essential to reduce the risk of pseudo-replication and information leakage. All images belonging to the same patient were assigned exclusively to one cross-validation fold; consequently, images from the same patient could not appear simultaneously in the training and validation sets. Thus, data separation was ensured at the patient level rather than by random image-level splitting. However, even with GroupKFold splitting, multiple correlated ROIs from the same patient may still limit patient diversity and may increase the possibility that the model learns patient-specific radiographic characteristics, acquisition-related patterns, or anatomical particularities rather than osteoporosis-specific features. Accordingly, the reported metrics should be interpreted as exploratory ROI-level performance under patient-level grouped validation rather than as fully independent image-level observations or definitive patient-level diagnostic performance.

### 2.8. Outcome Measures and Evaluation Metrics

The primary outcome was ROI-level discriminatory performance under patient-level grouped cross-validation between osteoporotic and non-osteoporotic classes within each ROI-specific experiment, evaluated within a patient-level grouped cross-validation framework. Model performance was assessed using accuracy, precision, recall, F1 score, and AUC. Metrics were calculated at the ROI/image level for each fold separately and subsequently averaged across all five folds.

Several ROIs could originate from the same patient; therefore, the reported metrics should not be interpreted as fully independent image-level observations. Rather, they represent ROI-level classification performance obtained under patient-level grouped validation, meaning that patient separation was maintained between training and validation folds. No fully aggregated patient-level probability analysis was performed as a primary outcome. Therefore, the results are interpreted as exploratory ROI-level model performance with patient-level separation between training and validation folds, rather than as definitive patient-level diagnostic performance.

### 2.9. Hypothesis Definition

Because this study was designed as an exploratory two-group image-classification study, the primary analytical objective was to describe discriminatory performance between osteoporotic and non-osteoporotic classes within ROI-specific model configurations [26]. The study was not designed as a confirmatory equivalence or non-inferiority trial. Therefore, no formal equivalence testing, non-inferiority testing, or Two One-Sided Tests (TOST) procedure was performed.

Although equivalence-based approaches may be conceptually useful for future studies investigating nondiscriminatory AI findings, such analyses require an a priori clinically justified equivalence margin. In the present pilot study, no such margin was prespecified. Therefore, nondiscriminatory or unstable model behavior was interpreted as limited classification performance under the evaluated experimental conditions, rather than as statistical evidence of equivalence, non-inferiority, or true radiographic indistinguishability between osteoporotic and non-osteoporotic bone.

### 2.10. Explainability Analysis

To assess model attention, gradient-weighted class activation mapping was used to generate visual explanations of class-relevant image regions [33]. Activation maps were generated for selected validation images from each fold and overlaid on the original ROI images. Gradient-weighted class activation mapping (Grad-CAM) was used here as an exploratory localization tool rather than as proof of mechanistic interpretability. The purpose of this analysis was to examine whether the model consistently attended to anatomically plausible regions across folds and samples. A lack of stable attention patterns was interpreted as supportive of limited feature consistency, while not being considered sufficient to prove radiographic equivalence.

In addition, Grad-CAM outputs were reviewed in a human-in-the-loop manner to assess whether model attention was located within anatomically plausible regions of the selected ROI rather than in obviously irrelevant image areas. This quality-control step was intended to reduce the risk that model predictions relied exclusively on visually implausible shortcuts. However, Grad-CAM inspection cannot prove causal feature use and cannot fully exclude patient-specific learning or shortcut learning. Therefore, the heatmap analysis was interpreted only as an exploratory plausibility check.

### 2.11. Statistical Analysis

Performance metrics were summarized descriptively across the five validation folds as mean ± standard deviation. Accuracy, F1 score, and area under the receiver operating characteristic curve (AUC) were additionally reported with exploratory 95% confidence intervals (CIs) calculated across the five fold-level metric values using the t-distribution. These CIs were used descriptively to illustrate fold-to-fold variability and should not be interpreted as formal population-level confidence intervals.

The age difference between the osteoporosis and control groups was statistically evaluated using Welch’s *t*-test because of the unequal group sizes and the possibility of unequal variances. The mean age difference between groups was reported to have a 95% confidence interval (CI), and the magnitude of the difference was additionally described using Cohen’s d. Because all included patients were female, sex was constant across the cohort and could not be evaluated as an independent confounding variable.

In addition, an exploratory metadata-only sensitivity analysis using available demographic metadata was performed to assess whether age alone showed a relevant association with the classification outcome. This analysis did not indicate a stable independent effect of age on the outcome and was therefore not included as a primary model component. Because the present study was designed as an exploratory supervised classification study, model performance was evaluated descriptively on the basis of fold-wise classification metrics. No formal equivalence test, non-inferiority analysis, or Two One-Sided Tests (TOST) procedure was performed because no clinically justified equivalence or non-inferiority margin had been defined a priori. Consequently, the reported analyses should be interpreted as descriptive and exploratory performance estimates, not as confirmatory evidence of equivalence to chance-level classification, non-inferiority, or equivalence to human interpretation.

All analyses and model development were performed in Python 3.13.5. Deep learning models were implemented using PyTorch 2.7.1, and cross-validation as well as performance metrics were calculated using scikit-learn 1.7.1. Image preprocessing and Grad-CAM visualization were performed using standard Python-based image-analysis libraries.

## 3. Results

### 3.1. Study Population and Dataset Composition

The study cohort comprised 51 patients, including 25 patients in the osteoporosis group and 26 patients in the control group. The osteoporosis group included patients with a confirmed diagnosis of osteoporosis, whereas the control group comprised patients without a documented history or diagnosis of osteoporosis. The mean age of the overall study population was 71.1 ± 9.1 years, with a mean age of 76.2 ± 8.0 years in the osteoporosis group and 66.2 ± 7.3 years in the control group. Overall, the cohort included 51 women and 0 men; the osteoporosis group comprised 25 women and 0 men, whereas the control group comprised 26 women and 0 men. The age range was 56.3–93.2 years in the overall cohort, 63.8–93.2 years in the osteoporosis group, and 56.3–87.1 years in the control group. Additional demographic and clinical characteristics are summarized in Table 1.

A total of 1784 radiographic samples were included across both experiments. Of these, 1682 images were assigned to the peri-implant ROI experiment and 102 images to the mental foramen ROI experiment. In the peri-implant dataset, 861 images originated from patients in the osteoporosis group and 821 images from patients in the control group. In the mental foramen dataset, 50 images originated from patients in the osteoporosis group and 52 images from patients in the control group.

The number of image crops per patient differed substantially between experiments. The peri-implant experiment included an average of approximately 33.0 ROIs per patient (1682 ROIs from 51 patients), whereas the mental foramen experiment included approximately 2.0 ROIs per patient (102 ROIs from 51 patients). Thus, the number of radiographic samples was considerably larger than the number of statistically independent patients, particularly in the peri-implant experiment. Because patient-specific crop counts were not uniformly distributed, this imbalance was considered when interpreting model performance and is discussed as a potential source of pseudo-replication and patient-specific feature learning.

In the peri-implant experiment, the radiographic acquisition period ranged from 22 November 2012 to 7 October 2024 for the osteoporosis group and from 5 September 2013 to 23 October 2024 for the control group. All datasets were partitioned using fivefold GroupKFold cross-validation, ensuring that all images from a given patient were assigned to a single fold and that no patient contributed images to both training and validation within the same fold. In each fold, the data were split at the patient level into approximately 80% training and 20% validation cases.

The osteoporosis group was significantly older than the control group. The mean age difference between groups was 10.0 years. Welch’s *t*-test showed a statistically significant age difference between the osteoporosis and control groups (t = 4.64, df = 48.20, *p* < 0.001), with a 95% CI for the mean difference of 5.67–14.34 years. The effect size was large (Cohen’s d = 1.30). Therefore, age was considered a potential confounding factor when interpreting model performance. However, an exploratory metadata-only sensitivity analysis using age did not indicate a stable independent effect of age on the classification outcome. Because all included patients were female, sex was constant across the cohort and could not contribute to outcome discrimination.

### 3.2. Peri-Implant ROI Experiment

Performance metrics for the peri-implant ROI experiment are summarized in Table 2. Across the five validation folds, accuracy ranged from 0.4870 to 0.7403, F1-score ranged from 0.5093 to 0.7945, and AUC ranged from 0.5948 to 0.8612. Mean performance across folds was 0.613 ± 0.107 for accuracy, 0.628 ± 0.120 for F1 score, and 0.713 ± 0.098 for AUC. The exploratory 95% CIs were 0.480–0.746 for accuracy, 0.480–0.777 for F1 score, and 0.591–0.835 for AUC. These broad intervals indicate substantial uncertainty and fold-to-fold variability.

Across all folds, training accuracy increased progressively over the course of training. In contrast, validation accuracy and validation F1-score fluctuated across epochs without showing a consistent upward trend. Training and validation loss curves demonstrated the expected separation during optimization, with lower loss values in the training set than in the validation set. Because no early stopping criterion was applied, the complete epoch-wise training and validation behavior could be observed.

**Figure 3:**
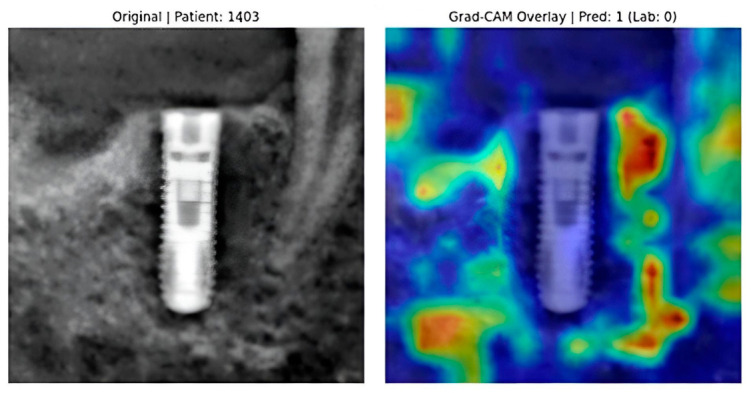
Grad-CAM visualization of a peri-implant region of interest. The grayscale image on the left shows the cropped peri-implant ROI surrounding a dental implant, and the Grad-CAM overlay on the right shows image regions contributing to the model prediction. In this example, the model predicted the osteoporotic class, whereas the reference label was non-osteoporotic. Heatmap colors indicate relative activation after normalization within the displayed image: blue/green represents lower activation, while yellow, orange, and dark red indicate stronger contribution to the predicted osteoporotic class. The strongest activations are located adjacent to the implant and in surrounding trabecular bone regions, where reduced or heterogeneous radiographic bone density may have influenced the model output. The Grad-CAM overlay should be interpreted only as an exploratory visualization of model attention and not as direct evidence of osteoporosis-specific bone loss.

Grad-CAM heatmaps were generated for validation images from all folds. In many samples, activation maps highlighted the implant neck and adjacent trabecular bone. However, the spatial distribution of activations varied between folds and also between samples within the same fold. In addition, some activation patterns extended into masked or clinically less relevant regions. A representative Grad-CAM visualization of a peri-implant ROI is shown in Figure 3.

### 3.3. Mental Foramen ROI Experiment

Performance metrics for the foramen ROI experiment are summarized in Table 3. Across the five validation folds, accuracy ranged from 0.5195 to 0.7792, F1-score ranged from 0.5488 to 0.8387, and AUC ranged from 0.5829 to 0.8610. Mean performance across folds was 0.701 ± 0.111 for accuracy, 0.713 ± 0.107 for F1 score, and 0.744 ± 0.115 for AUC. The exploratory 95% CIs were 0.563–0.839 for accuracy, 0.580–0.845 for F1 score, and 0.601–0.888 for AUC. Although the mental foramen ROI showed numerically higher mean performance than the peri-implant ROI, the confidence intervals remained broad.

Across folds, training accuracy increased steadily over epochs. Validation metrics showed moderate variability between epochs and between folds. Compared with accuracy and F1-score, validation AUC appeared relatively more stable over the course of training, although fold-to-fold differences remained present.

### 3.4. Comparison of ROI-Specific Performance

A comparison of the mean performance metrics for both experiments is shown in Table 4. The foramen ROI classifier achieved higher mean values for accuracy, F1-score, and AUC than the peri-implant ROI classifier.

Overall, fold-to-fold variability was observed in both experiments. However, the foramen ROI showed numerically higher average performance across all reported metrics. No consistent epoch-wise convergence pattern clearly differentiated the two ROI-specific classification tasks.

### 3.5. Aggregated Statistical Visualization

**Figure 4:**
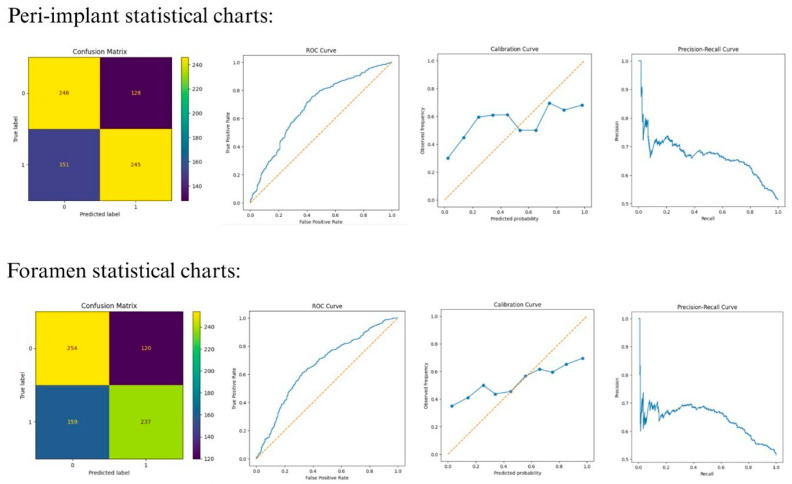
Aggregated statistical evaluation of the peri-implant and foramen ROI classification experiments. The upper row shows the peri-implant ROI results and the lower row the foramen ROI results, including the confusion matrix, ROC curve, calibration curve, and precision–recall curve. In both experiments, ROC curves indicated performance above chance, whereas calibration curves showed deviations from ideal calibration. Overall, the foramen ROI experiment showed slightly better performance than the peri-implant ROI experiment, consistent with Table 4.

Figure 4 summarizes the aggregated statistical outputs for both ROI-specific experiments. In the upper row, corresponding to the peri-implant experiment, the confusion matrix showed a moderate balance between correctly classified osteoporotic and non-osteoporotic cases, with persistent false-positive and false-negative predictions. The receiver operating characteristic (ROC) curve indicated discriminatory performance above chance, consistent with the fold-averaged AUC values. The calibration curve showed visible deviations from the diagonal reference line, indicating imperfect agreement between predicted probabilities and observed frequencies. The precision–recall curve demonstrated moderate precision across a broad range of recall values, with a gradual decline at higher recall levels.

In the lower row, corresponding to the foramen experiment, the confusion matrix similarly demonstrated both correct classifications and remaining misclassifications. The ROC curve showed slightly improved overall separation compared with the peri-implant experiment, in line with the numerically higher mean performance metrics observed for this ROI. The calibration curve again deviated from the ideal diagonal, suggesting that probability estimates were not perfectly calibrated. The precision–recall curve showed a comparable overall pattern, with moderate precision across increasing recall levels and declining precision toward the highest recall range.

### 3.6. Additional Observations

In the peri-implant ROI experiment, the delta-days variable was incorporated as an additional metadata input. Because no separate metadata-only or ablation analysis was performed, the isolated quantitative contribution of this variable to final model performance could not be determined.

The reported classification metrics were calculated at the ROI/image level within patient-level grouped validation folds. Therefore, the present results do not represent fully aggregated patient-level diagnostic predictions. A patient-level aggregation of predicted probabilities was not performed as a primary analysis because the peri-implant dataset contained a variable number of ROIs per patient, which could have introduced additional weighting effects depending on the number of available crops per individual. Instead, patient-level independence was addressed at the cross-validation stage by assigning all ROIs from the same patient to the same fold.

## 4. Discussion

This study evaluated whether osteoporosis-related mandibular bone differences can be detected on dental radiographs by deep learning when analysis is restricted to 2 biologically distinct regions of interest: peri-implant bone and the mental foramen region. Under otherwise comparable training, preprocessing, and grouped cross-validation conditions, the foramen model yielded numerically higher mean validation performance than the peri-implant model, whereas both approaches showed clear instability across folds. Thus, the present findings suggest that region selection influences model behavior, but neither ROI provided sufficiently consistent validation results to support reliable ROI-level classification performance under patient-level grouped validation. This interpretation is important in the context of dental AI research, where high performance has been reported for more visually overt tasks such as caries, periapical lesion, periodontal bone loss, and cone-beam computed tomography (CBCT)-supported diagnosis, but performance remains task-dependent and strongly influenced by dataset characteristics, label quality, and anatomic target definition [1,2,3,4,6].

The biological rationale for testing both ROIs remains plausible. Osteoporosis affects bone quality, trabecular organization, and remodeling capacity, and impaired regenerative responses in osteoporotic bone have been demonstrated experimentally [9,10]. Accordingly, peri-implant bone may theoretically reflect altered healing dynamics, whereas the foramen region may better reflect systemic mandibular architecture independent of surgical intervention. Previous dental radiographic research has shown that mandibular cortical and trabecular features, particularly in the mental foramen region on panoramic radiographs, may aid osteoporosis triage screening [7,8,11,12]. However, these prior studies do not establish that the cropped periapical foramen ROI used here should function as a “gold standard.” Rather, they support the expectation that the foramen region may contain more accessible systemic information than peri-implant bone. The slightly stronger foramen results in the present study are therefore directionally compatible with prior screening literature, but the remaining variability prevents any definitive claim of robust detectability [8,14].

A relevant potential confounding factor was the age imbalance between groups. The osteoporosis group was significantly older than the control group, and age itself is known to affect mandibular bone structure, trabecular organization, cortical thickness, and radiographic texture. Therefore, the model may have learned age-associated radiographic patterns rather than osteoporosis-specific features. This possibility is particularly relevant for subtle dental radiographic classification tasks, where age-related bone changes and systemic disease-related bone alterations may overlap. However, an exploratory metadata-only sensitivity analysis using age did not indicate a stable independent effect of age on the classification outcome. In addition, sex could not act as a discriminating metadata variable because all included patients were female. Nevertheless, because no formal age-adjusted image-based model or age-stratified sensitivity analysis was performed, residual confounding by age cannot be excluded. Future studies should use larger cohorts with closer age matching or include age-adjusted modeling to better separate osteoporosis-specific radiographic features from age-related bone changes.

A central message of this study is methodological. Failure of a model to discriminate is not equivalent to proof that no radiographic difference exists. This principle is well established in classical inference, where absence of evidence cannot be interpreted as evidence of absence [15]. In machine learning, that distinction becomes even more critical because performance is shaped by many nonbiologic factors, including sample size, fold composition, preprocessing, architecture, uncertainty, and hidden confounding [16,18,19]. Thus, the unstable validation metrics observed here indicate limited extractable signal under the present conditions, but they do not justify a formal conclusion of radiographic equivalence between groups [21].

The training dynamics reinforce this interpretation. In both experiments, training performance improved steadily, whereas validation metrics fluctuated, a pattern more consistent with fitting restricted training structure than with learning a stable and generalizable disease signature. Such behavior is compatible with weak signal, small-sample instability, or shortcut learning rather than clinically meaningful discrimination [17,34,35]. The Grad-CAM findings should also be interpreted cautiously. Although Grad-CAM can provide exploratory localization of attention [33], saliency methods in medical imaging have recognized limitations and may fail to localize subtle or clinically relevant features reliably [29,30]. In the present setting, the inconsistent activation patterns are therefore supportive of limited feature stability, but they are not mechanistic proof of absence of disease-specific information.

These findings also argue for a more cautious and inference-oriented framework for interpreting negative or nondiscriminatory AI results in radiology. Reporting checklists and evaluation frameworks such as the Checklist for Artificial Intelligence in Medical Imaging (CLAIM), broader guidance for reading clinical AI studies, editorial recommendations for radiologic AI, and the Appraisal of Artificial Intelligence Studies for Clinical Decision Support (APPRAISE-AI) emphasize transparency, methodological rigor, and reproducibility [22,23,24,25]. However, rigorous reporting alone does not resolve the inferential question of whether weak discrimination reflects true biological similarity, insufficient experimental sensitivity, limited sample size, hidden confounding, or model instability.

The present study should therefore be regarded as exploratory with respect to equivalence-oriented interpretation. Although concepts such as equivalence testing, non-inferiority testing, and Two One-Sided Tests (TOST) may provide useful frameworks for future studies of nondiscriminatory AI performance, these approaches were not implemented in the present analysis [26,27,28]. A formal equivalence or non-inferiority analysis would require a clinically justified and prospectively defined margin, for example an acceptable difference in AUC relative to a predefined reference standard or expert-level human interpretation. In the present pilot study, such a margin was not prespecified, and the available sample size was limited at the patient level [20]. Therefore, the present findings should not be interpreted as evidence of equivalence to chance-level classification, equivalence to human interpretation, or proof of radiographic indistinguishability. Instead, they indicate that the evaluated models did not demonstrate stable and externally validated discriminatory performance under the present experimental conditions [36].

This study has limitations. The cohort was small at the patient level, which increases uncertainty of cross-validation estimates and may inflate fold-to-fold variability [21]. Although patient-level GroupKFold validation was used to prevent direct information leakage between training and validation sets, the large number of image crops relative to the limited number of patients introduces a risk of pseudo-replication. Multiple ROIs from the same patient are not statistically independent, and the effective sample size is therefore closer to the number of patients than to the number of image crops. This may limit patient diversity and may allow the model to learn patient-specific radiographic characteristics, acquisition-related patterns, or anatomical particularities rather than osteoporosis-specific features [17,21,31,32]. In addition, the reported performance metrics were calculated at the ROI/image level and not as fully aggregated patient-level predictions. Although patient-level GroupKFold splitting prevented images from the same patient from appearing in both training and validation sets, the absence of a separate patient-level probability aggregation limits direct interpretation of the results as patient-level diagnostic performance.

A major limitation is the absence of external validation. The present investigation should be considered a single-center pilot study based entirely on internally available retrospective radiographic data. Although grouped cross-validation was used to obtain an internal estimate of model performance, no independent external test cohort from another institution, imaging system, patient population, or acquisition protocol was available. Therefore, the reported results cannot be interpreted as evidence of external robustness or clinical generalizability [16,21,23,24,25]. This limitation is particularly relevant because both ROI-specific experiments showed substantial fold-to-fold variability, indicating that model performance was sensitive to the composition of the validation folds.

Human-in-the-loop ROI selection and Grad-CAM heatmap inspection were used to assess whether model attention was located within anatomically plausible regions and to reduce reliance on visually implausible shortcuts. However, these procedures cannot fully exclude patient-specific learning or shortcut learning. In addition, Grad-CAM should be interpreted only as an exploratory plausibility tool, because saliency methods do not prove causal feature use or mechanistic interpretability [29,30,33]. No formal equivalence test, ablation study, metadata-only comparison, or unrelated baseline ROI was performed. Consequently, the incremental role of delta-days and the relative interpretability of “higher,” “intermediate,” or “chance-like” ROI performance could not be quantified directly. In addition, the mental foramen findings should not be overgeneralized to all mandibular screening strategies, because the present design used cropped periapical ROIs rather than established panoramic screening workflows [8,11,12,13,14]. External validation in larger, independent, preferably multicenter cohorts is required before any conclusion regarding diagnostic applicability, robustness, or generalizability can be drawn. Therefore, the results should be interpreted as evidence of limited and unstable discriminatory performance under the present experimental conditions, rather than as definitive evidence for or against the presence of osteoporosis-related radiographic differences.

## 5. Conclusions

Deep learning applied to peri-implant and mental foramen ROIs showed only modest and unstable ability to distinguish osteoporotic from non-osteoporotic patients on dental radiographs. Although the mental foramen region achieved numerically higher mean validation performance than the peri-implant region, both approaches showed substantial fold-to-fold variability and broad uncertainty around the performance estimates. Therefore, neither ROI yielded sufficiently consistent evidence to support reliable and generalizable ROI-level classification performance, and the results should not be interpreted as individual patient-level screening performance.

The present findings should not be interpreted as proof of radiographic equivalence between osteoporotic and non-osteoporotic bone. No formal equivalence or non-inferiority testing was performed because no clinically justified margin had been defined a priori. Rather, they indicate that, under the present experimental conditions, no robust and generalizable osteoporosis-related radiographic signature could be established. This distinction is important because nondiscriminatory AI performance may reflect limited sample size, ROI heterogeneity, model instability, hidden confounding, age-related confounding, patient-specific feature learning, or insufficient radiographic signal rather than true biological indistinguishability.

Because this was a single-center pilot study without an independent external test set, the present findings should not be interpreted as evidence of clinical robustness or generalizability. Future studies should combine multi-ROI modeling with larger patient-level datasets, multicenter external validation, predefined clinically justified equivalence or non-inferiority margins, formal equivalence testing, power analysis, age-adjusted or age-matched study designs, and expert-anchored interpretation of explainability outputs. Such approaches are needed to assess nondiscriminatory AI findings with the same inferential rigor expected in medical imaging research.

## Figures and Tables

**Table 1:** Demographic and clinical characteristics of the study population.

Characteristic	Overall Cohort (*n* = 51)	Osteoporosis Group (*n* = 25)	Control Group (*n* = 26)
Age, mean ± SD (years)	71.1 ± 9.1	76.2 ± 8.0	66.2 ± 7.3
Age range (years)	56.3–93.2	63.8–93.2	56.3–87.1
Female, *n*	51	25	26
Male, *n*	0	0	0
Peri-implant images, *n*	1682	861	821
Mental foramen images, *n*	102	50	52
Radiograph acquisition period *	22 November 2012–23 October 2024	22 November 2012–7 October 2024	5 September 2013–23 October 2024

* Applicable to the peri-implant experiment only.

**Table 2:** Peri-implant ROI classification performance.

Fold	Accuracy	F1-Score	AUC
0	0.5519	0.5306	0.5948
1	0.7078	0.7945	0.7333
2	0.5779	0.6061	0.7085
3	0.7403	0.7015	0.8612
4	0.4870	0.5093	0.6667

**Table 3:** Foramen ROI classification performance.

Fold	Accuracy	F1-Score	AUC
0	0.5195	0.5488	0.5829
1	0.7727	0.8387	0.7828
2	0.6688	0.7411	0.6690
3	0.7662	0.7500	0.8251
4	0.7792	0.6852	0.8610

**Table 4:** Comparison of experiment metrics averaged across folds.

ROI	Accuracy (Mean)	F1-Score (Mean)	AUC (Mean)
Peri-implant	0.6130	0.6284	0.7129
Foramen	0.7013	0.7128	0.7442

## Data Availability

The data presented in this study are available on reasonable request from the corresponding author. The data are not publicly available due to privacy, ethical, and legal restrictions related to patient radiographic data.

## Abbreviations

The following abbreviations are used in this manuscript:

**Abbreviation**	**Definition**
AI	Artificial intelligence
APPRAISE-AI	Appraisal of Artificial Intelligence Studies for Clinical Decision Support
AUC	Area under the receiver operating characteristic curve
CBCT	Cone-beam computed tomography
CLAIM	Checklist for Artificial Intelligence in Medical Imaging
Grad-CAM	Gradient-weighted class activation mapping
MCML	Munich Center for Machine Learning
NonOP	Non-osteoporotic
OP	Osteoporotic
OPTG	Orthopantomogram
PRISMA	Preferred Reporting Items for Systematic Reviews and Meta-Analyses
ROI	Region of interest
ROC	Receiver operating characteristic
SD	Standard deviation
TOST	Two one-sided tests
TUM	Technical University of Munich
